# Thoracic skeletal muscle index is effective for CT-defined sarcopenia evaluation in patients with head and neck cancer

**DOI:** 10.1007/s00405-023-08162-y

**Published:** 2023-08-12

**Authors:** Belinda Vangelov, Robert Smee, Daniel Moses, Judith Bauer

**Affiliations:** 1 Department of Radiation Oncology, Nelune Comprehensive Cancer Centre, Prince of Wales Hospital and Community Health Services, Level 1, Bright Building, Avoca St, Randwick, NSW 2031 Australia; 2 School of Clinical Medicine, Randwick Campus, Faculty of Medicine and Health, University of New South Wales, Randwick, NSW 2031 Australia; 3 Department of Radiation Oncology, Tamworth Base Hospital, Tamworth, NSW 2340 Australia; 4 Graduate School of Biomedical Engineering, University of New South Wales, Randwick, NSW 2031 Australia; 5 Department of Radiology, Prince of Wales Hospital and Community Health Services, Randwick, NSW 2031 Australia; 6 Department of Nutrition, Dietetics and Food, School of Clinical Sciences, Monash University, Clayton, VIC 3168 Australia

**Keywords:** Head and neck cancer, Sarcopenia, Skeletal muscle, Thoracic muscle, Weight loss, Computed tomography

## Abstract

**Purpose:**

Computed tomography (CT)-defined sarcopenia, as a measurement of low skeletal muscle (SM), is a poor prognostic indicator in patients with head and neck cancer (HNC), independent of weight or nutritional status. We used SM measures at the second thoracic vertebra (T2) to determine T2-SM index (SMI) thresholds for sarcopenia, and investigate the impact of low T2-SMI on overall survival (OS), and weight loss during radiotherapy (RT).

**Methods:**

Adult patients with newly diagnosed HNC with a diagnostic PET–CT or RT planning CT scan were included. SM was analysed at T2 and a model applied to predict SM at L3. T2-SMI thresholds for sarcopenia were established with predicted measures, stratified by BMI and sex. Impact of sarcopenia and low T2-SMI on OS and weight loss during RT was investigated.

**Results:**

A total of 361 scans were analysed (84% males, 54% oropharynx tumours). Sarcopenia was found in 49%, demonstrating worse OS (*p* = 0.037). T2-SMI cutoff values were: females—74 cm^2^/m^2^ [area under the curve (AUC): 0.89 (95%CI 0.80–0.98)], males (BMI < 25)—63 cm^2^/m^2^ [AUC 0.93 (95%CI 0.89–0.96)], males (BMI ≥ 25)—88cm^2^/m^2^ [AUC 0.86 (95%CI 0.78–0.93)]. No difference in OS with T2-SMI categories. Lowest T2-SMI quartile of < 63 cm^2^/m^2^ demonstrated worse OS (*p* = 0.017). Weight loss during RT was higher in patients; who were not sarcopenic (6.2% vs 4.9%, *p* = 0.023); with higher T2-SMI (6.3% vs 4.9%, *p* = 0.014) and; in the highest quartiles (3.6% vs 5.7% vs 7.2%, *p* < 0.001).

**Conclusions:**

These T2-SMI thresholds are effective in assessing CT-defined sarcopenia in HNC. Further assessment of clinical application is warranted.

**Supplementary Information:**

The online version contains supplementary material available at 10.1007/s00405-023-08162-y.

## Introduction

The incidence of head and neck cancer (HNC) worldwide is increasing, with more than 650,000 cases annually and 380,000 deaths [1]. In Australia, in 2019, there were over 5000 reported cases [2]. Malnutrition has been reported in up to 70% of patients with HNC [3–6], manifesting as a multifactorial syndrome due to a combination of inadequate oral intake and metabolic derangements, caused by the tumour itself, and/or the impact of treatment-related toxicities [7, 8]. One of the phenotypic diagnostic criteria for malnutrition, as recommended by the Global Leadership Initiative on Malnutrition (GLIM) is reduced skeletal muscle mass [9], with methods of evaluation making opportunistic use of diagnostic computed tomography (CT) images in patients with cancer. Low muscle mass, quantified with CT images as skeletal muscle index (SMI), is known as CT-defined sarcopenia, the prevalence of which has been demonstrated to be a poor prognostic indicator, independent of weight or nutritional status [10–12], especially in patients with HNC [13, 14]. The incorporation of body composition measures, especially skeletal muscle mass assessment, in patients with HNC allows for more robust nutritional screening at the time of diagnosis.

The most widely used method for CT skeletal muscle analysis is assessment of the cross-sectional area (CSA) of an axial slice at the level of the third lumbar vertebra (L3), as muscle at this level has been shown to best represent whole body measures [15, 16]. However, abdominal scans with the L3 level visible may not be readily available in patients with HNC. Alternative anatomical landmarks have been used in many recent studies, some yet to be validated, and with varying results. The most commonly used alternative in HNC is the third cervical vertebra (C3), with some researchers also choosing thoracic options [17]. Many of these studies have formulated prediction models to estimate L3-CSA using measures at other vertebral levels. Our group has discovered recently that the most commonly used prediction model applying CSA measures at C3 demonstrated weak agreement with actual measures in our Australian cohort of majority overweight or obese patients with HNC [18]. Positioning of the neck, the presence of tumour, and concern surrounding the appropriateness of muscles in the neck to predict musculature in the abdomen, or whether muscle wasting occurs in similar proportions, are all issues that caution the use of muscle measures at this level [19]. Assessment of muscle at the thoracic level may be more feasible due to the larger muscle groups present that may be more susceptible to muscle wasting, and we have previously shown that measures taken at the second thoracic level (T2) correlate well to measures at L3 [20]. Pai et al. measured adipose tissue and skeletal muscle at T2 and suggested cutoff values based on sex-specific median values in a Taiwanese population, finding that skeletal muscle measures have no significant impact on overall survival (OS) [21]. However, theses ranges are very population-specific and not translatable to our Australian cohort. At present, there are no HNC-specific cutoff values for sarcopenia that are applicable across populations.

The aim of this study was to determine SMI cutoff values for sarcopenia using T2-CSA measures in our cohort of patients with HNC, and to determine the association between T2-measured low SMI and OS, and weight loss during treatment.

## Materials and methods

This is an Ethics approved (2019/ETH13149), single-institution, retrospective cohort study investigating CT-defined sarcopenia in all newly diagnosed adult patients (≥ 18 years) with HNC who presented to our facility between January 2005 and February 2022. Inclusion criteria were patients with; pathology-confirmed squamous cell carcinoma of the larynx, hypopharynx, oropharynx, nasopharynx or oral cavity and a diagnostic PET–CT scan or radiotherapy (RT) planning CT scan. Patients were excluded if they had history of a previous cancer diagnosis, or had an unusable CT scan. “Unusable” CT scans were classified as those images with low resolution, overt skewing of the humeral heads at the level of T2, or if any musculature (such as the deltoids) were outside the frame of the scan. Patient baseline and treatment characteristics were retrieved from electronic and written patient records. Weights and heights were recorded at the time of PET–CT scan or at the time of attendance to the head and neck clinic. In patients who completed treatment, weights were also recorded in the final week of radiotherapy and percentage weight loss calculated.

### Skeletal muscle analysis

All suitable PET–CT scans and RT planning CT scans were anonymised and assessed for skeletal muscle CSA at the level of T2 via manual delineation by a single observer (trained in the Alberta Protocol with a 1.1% interrater variation achieved) (BV) with landmarking accuracy supervised by a Senior Radiologist (DM). Slice-O-Matic Version 5.0 (Tomovision, Montreal, Canada) was used for muscle delineation, with skeletal muscle identified with standard Hounsfield Units (HU) of − 29 to + 150 HU [22, 23]. Landmarking of the axial slice to be analysed at T2 utilised previously described methodology [20], where the vertebral level was selected by scrolling from a cephalad to caudal direction from the top of the second thoracic vertebra, four slices from the top, in scan slices of 3 mm thickness. All large muscle groups visible in the scan were tagged and included: the sternocleidomastoid, pectoralis major and minor, deltoid, trapezius, subscapularis, rhomboid major, infraspinatus, splenius cervicis, and multifidus muscles, with smaller, less clearly defined muscles not analysed.

Skeletal muscle at L3 was also analysed in patients who had a PET–CT to test T2-SMI values against actual L3-SMI measures and compare to cutoff values determined with predicted L3-SMIs. CSA at L3 was measured using previously defined methodology [22, 23].

### Sacropenia assessment

Skeletal muscle T2-CSA was recorded and the previously described prediction model[20] was applied to convert the values into predicted values at L3;

$${\text{L3 - CSA}}\;({\text{cm}}^{2} ) = 174.15 + [0.212 \, \times {\text{T2 - CSA}}\;({\text{cm}}^{2} )] - [40.032 \times {\text{sex}}] - [0.928 \times {\text{age}}\;({\text{years}})] + [0.285 \times {\text{weight}}\;({\text{kg}})]$$

(For Sex use: 1 for male, 2 for female)

These predicted L3-CSA measures were normalised for stature and converted into SMI for each patient (cm^2^/height^2^). Patients were categorised by body mass index (BMI, kg/m^2^) as: underweight (BMI < 20.0), healthy weight (BMI 20.0–25.0), overweight (BMI 25.0–29.9) or obese (BMI ≥ 30.0). BMI measures were used to categorically classify patients as being sarcopenic as per previously defined BMI and sex-specific thresholds for females of SMI < 41 cm^2^/m^2^, and in males < 43 cm^2^/m^2^ (if underweight or healthy weight) and < 53 cm^2^/m^2^ (if overweight or obese) [24].

T2-CSA was also normalised for stature and converted to a measure of T2-SMI. The T2-SMI measures were used to determine corresponding cutoff values for sarcopenia classification. The whole cohort was used for T2-SMI cutoff analysis.

### Weight loss during radiotherapy

A sub-set of the cohort had completed radiotherapy (± other modalities) with curative intent and were included for weight loss analysis. Patients who did not complete prescribed treatment modality were excluded. The outcome measure was percentage weight loss during treatment. Total percentage weight loss was determined by subtracting the patients’ weight in the final week of radiotherapy from their initial weight, and converting to a percentage value. Percentage weight loss was compared amongst those with sarcopenia and those with low T2-SMI. Critical weight loss (CWL) was defined as a loss of ≥ 5%. The insertion of feeding tubes was noted and investigated in terms of T2-SMI category and CWL. Prophylactic tubes were defined as those inserted prior to treatment and reactive inserted during or post-RT completion.

### Survival

OS was defined as death from any cause and was calculated from the date of diagnostic or radiotherapy planning scan until date of death. Patients were censored if they were alive at the last date of follow-up. Comparison of sarcopenia status, T2-SMI cutoff values, and T2-SMI quartiles were investigated for association with OS.

### Statistical analysis

All analyses were performed using SPSS Statistics software package, Version 27.0 (IBM, Armonk, NY), with statistical significance set at *p* < 0.05 and all *p* values were two-sided. Descriptive continuous variable statistics are presented as mean ± standard deviation (SD) and normality determined using Shapiro–Wilk test. All categorical data are expressed as frequencies and percentages. Receiver Operator Characteristic (ROC) tests were performed on three groups (females, males BMI < 25 kg/m^2^ and males BMI ≥ 25 kg/m^2^), to determine sex and BMI-specific T2-SMI cutoff values based on sarcopenia status. The area under the curve (AUC), sensitivity, and specificity of the cutoff values are described, and the Youden index was used to identify the optimal T2-SMI cutoff values for each patient group. Patients were classified as having a “low” T2-SMI below the cutoff. T2-SMI quartile values were also compared to determine the cutoff point with the highest risk of sarcopenia, i.e., those with the lowest T2-SMI measures. This was also tested using the actual L3-SMI measurements in the sub-set of patients with a PET–CT scan (*n* = 111). Chi-squared test was applied to determine the sensitivity and specificity of the T2-SMI measures compared to sarcopenia. Clinical and disease predictors of sarcopenia were analysed using binary logistic regression, with multivariate models applying both T2-SMI and T2-SMI cutoff categories separately as potential predictors.

Weight loss percentages in patients with or without sarcopenia and those with low T2-SMI or not were compared using independent samples *T* tests. One-way ANOVA was used to investigate percentage weight loss differences between T2-SMI quartiles. Kaplan–Meier curves were used to visualise OS based on sarcopenia status and T2-SMI, and compared using the log-rank test. Univariate and multivariate analysis was conducted using Cox regression modelling to investigate the association of baseline characteristics on OS. The backward conditional method was applied, with a significance of *p* < 0.2 as criteria for variable inclusion in the multivariate model. Hazard ratios and 95% confidence intervals (CI) are provided.

## Results

A total of 530 patients had a diagnostic PET–CT scan or a radiotherapy planning scan between 2005 and 2022, of which 414 had a usable T2 axial slice. Fifty-three patients were excluded due to skewed shoulders or missing heights. Therefore, a total of 361 patient scans were included in this study, with this cohort used for T2-SMI cutoff analysis. Only 335 patients had completed radiotherapy (± other modalities), and were used for outcome investigations. Patient characteristics are displayed in Table 1. The majority of patients were male (84%), the largest tumour group was oropharynx carcinoma (54%) and of those patients who completed treatment (*n* = 335), the majority had concurrent chemoradiotherapy (51%).

**Table 1:** Patient demographics

Characteristic	Whole population (*n* = 361) (%)	Patients who completed treatment (*n* = 335) (%)
Sex		
Male	303 (84)	280 (84)
Female	58 (16)	55 (16)
Age (years)		
Mean (SD)	60.5 (11.1)	60.1 (11.1)
Tumour site		
Larynx	60 (17)	53 (16)
Oropharynx	194 (54)	187 (56)
Nasopharynx	41 (11)	36 (11)
Hypopharynx	16 (5)	15 (4)
Oral Cavity	44 (11)	40 (12)
Unknown primary	6 (2)	4 (1)
Tumour stage		
Tis	3 (1)	3 (1)
T1	106 (29)	96 (28)
T2	101 (28)	98 (29)
T3	87 (24)	82 (25)
T4	58 (16)	52 (16)
Tx	6 (2)	4 (1)
Nodal stage		
N0	98 (27)	92 (27)
N1	97 (27)	88 (26)
N2	145 (40)	136 (41)
N3	21 (6)	19 (6)
Treatment modality		
RT only	–	75 (22)
RT + surgery	–	90 (27)
CRT	–	170 (51)
BMI category (kg/m^2^)		
Underweight (BMI < 20)	23 (6)	19 (6)
Normal weight (BMI ≥ 20 < 25)	108 (30)	99 (30)
Overweight (BMI ≥ 25 < 30)	147 (41)	138 (41)
Obese (BMI > 30)	83 (23)	79 (23)
Sarcopenia		
Yes	175 (48)	161 (48)
No	186 (52)	174 (52)
Sarcopenic obesity		
Yes	113 (65)	107 (67)
No	62 (35)	54 (33)

*SD* standard deviation, *RT* radiotherapy, *CRT* concurrent chemoradiotherapy, *BMI* body mass index

**Fig. 1:**
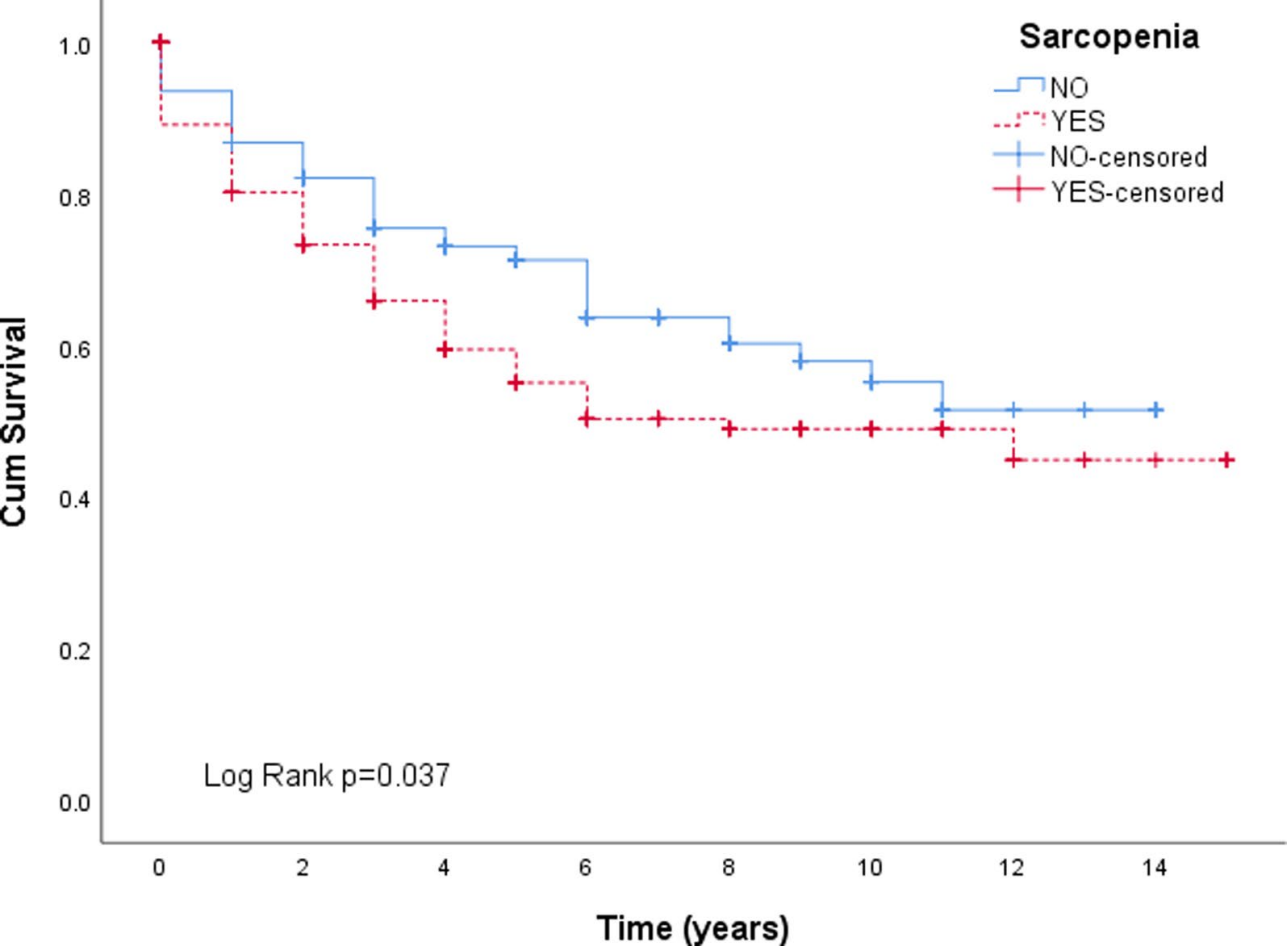
Overall survival with sarcopenia status

A total of 175 (49%) patients were sarcopenic using the prediction model for determining L3-CSA, and application of the previously described thresholds [24]. Sixty-five percent (*n* = 113) of these patients had sarcopenic obesity. Kaplan–Meier survival curves showed patients who were sarcopenic at diagnosis had worse OS (Log Rank *p* = 0.037) (Fig. 1), with 1-year survival rates of 81% vs 87%, and 5-year rates of 62% vs 76% when compared to those who were not sarcopenic. Median survival was 8.5 vs 9.4 years (95% CI 7.5–9.6).

**Fig. 2:**
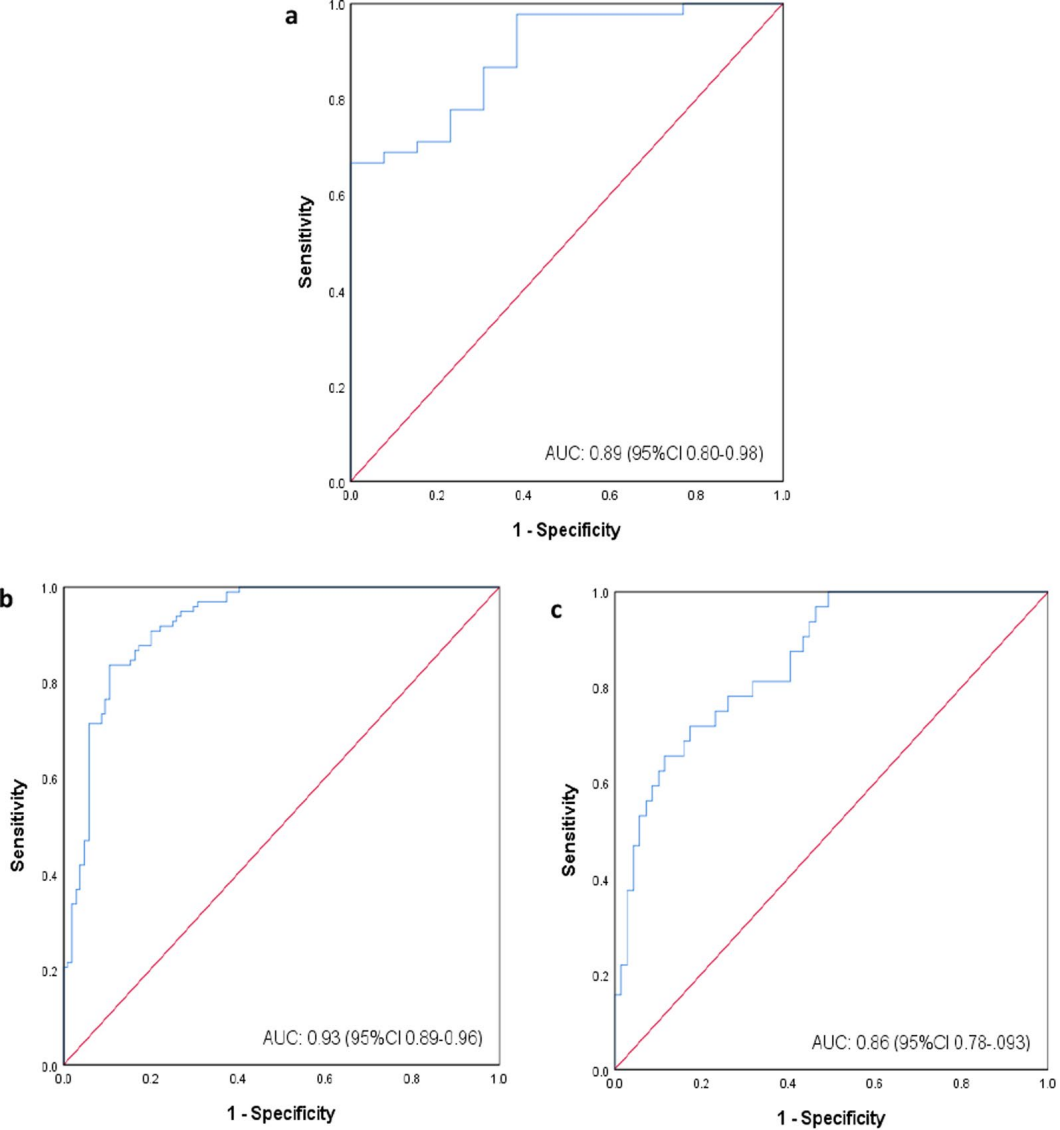
ROC curves for T2-SMI cutoff values based on sarcopenia classification. **a** Females, **b** males, BMI < 25 kg/m^2^M, **c** males, BMI ≥ 25 kg/m^2^

The cutoff values for low T2-SMI were categorised into three sex and BMI-specific groups. The ROC curves are displayed in Fig. 2, with all AUC values > 0.85 (considered to be good, and > 0.9 excellent). The T2-SMI cutoff values were; females (regardless of BMI), − 74 cm^2^/m^2^, and males: BMI < 25 kg/m^2^—63 cm^2^/m^2^ and BMI ≥ 25 kg/m^2^—88 cm^2^/m^2^. The sensitivity and specificity values with confidence intervals (CI) are shown in Table 2. Application of these cutoff values found that 181 (50%) patients had low T2-SMI. When patients were categorised as high or low T2-SMI, the comparison to corresponding sarcopenia diagnosis demonstrated a sensitivity of 80% and specificity of 82%. Testing of cutoff values using actual measures at L3 in patients with a PET–CT determined similar values to predicted measure (supplemental material, Fig. 4). Low T2-SMI was found to be a predictor of sarcopenia on multivariate logistic regression analysis (HR 67.28, CI 27.59–164.08, *p* < 0.001) along with sex (female, HR 10.27, CI 3.65–28.92, *p* < 0.001) and increasing age (HR 1.17, CI 1.11–1.22, *p* < 0.001) (Table 3). In separate analysis with the continuous T2-SMI variable, a lower T2-SMI value increased the risk of being sarcopenic (HR 0.93, CI 0.91–0.95, *p* < 0.001).

**Table 2:** T2-SMI cutoff ROC curve results for sarcopenia risk

	T2-SMI Cutoff (cm^2^/m^2^)	AUC	Sensitivity (%)	Specificity (%)	95% CI	*p* value
Females	74	0.89	78	77	0.80–0.98	**< 0.001**
Males (BMI < 25)	63	0.86	78	71	0.78–0.93	**< 0.001**
Males (BMI ≥ 25)	88	0.93	87	84	0.89–0.96	**< 0.001**

All significant values are in bold (*p* < 0.05)

*SMI* skeletal muscle index, *AUC* area under the curve, *CI* confidence interval, *BMI* body mass index

**Table 3:** Predictors of sarcopenia in patients who completed RT

Predictors	Sarcopenia				
	*n*	Univariate		Multivariate	
		HR (95% CI)	*p* value	HR (95% CI)	*p* value
Sex					
Male	280	Ref		Ref	
Female	55	4.31 (2.21–8.38)	** < 0.001**	10.27 (3.65–28.92)	**< 0.001**
Age (years)		1.08 (1.06–1.11)	** < 0.001**	1.17 (1.11–1.22)	**< 0.001**
Tumour site					
Larynx	53	Ref		Ref	
Oropharynx	187	0.95 (0.52–1.75)	0.432	1.46 (0.57–3.71)	0.43
Nasopharynx	36	0.32 (0.13–0.81)	**0.016**	0.28 (0.07–1.21)	0.088
Hypopharynx	15	3.85 (0.97–15.23)	0.055	2.07 (0.29–15.00)	0.47
Oral cavity	40	0.87 (0.38–1.98)	0.742	0.38 (0.10–1.40)	0.145
Tumour stage					
T1	96	Ref			
T2	98	1.28 (0.73–2.26)	0.386		
T3	82	1.23 (0.68–2.23)	0.488		
T4	52	1.23 (0.63–2.42)	0.545		
Nodal stage					
N0	92	Ref		Ref	
N1	88	0.49 (0.27–0.89)	**0.018**	0.75 (0.27–2.09)	0.576
N2	136	0.49 (0.89–0.84)	**0.010**	0.89 (0.34–2.36)	0.81
N3	19	0.58 (0.21–1.56)	0.28	1.17 (0.20–6.82)	0.865
Pre-treatment weight loss					
No	220	Ref			
Yes	115	0.97 (0.62–1.52)	0.892		
T2-SMI cutoff categories*					
Mod–high T2-SMI	162	Ref		Ref	
Low T2-SMI	173	16.44 (9.57–28.25)	**< 0.001**	67.28 (27.59–164.08)	**< 0.001**
T2-SMI (continuous)*		0.94 (0.92–0.95)	**< 0.001**	0.93 (0.91–0.95)	**< 0.001**

All significant values are in bold (*p* < 0.05)

*HR* hazard ratio, *CI* confidence interval, *SMI* skeletal muscle index

*Tested separately in logistic regression models independently with other variables

**Fig. 3:**
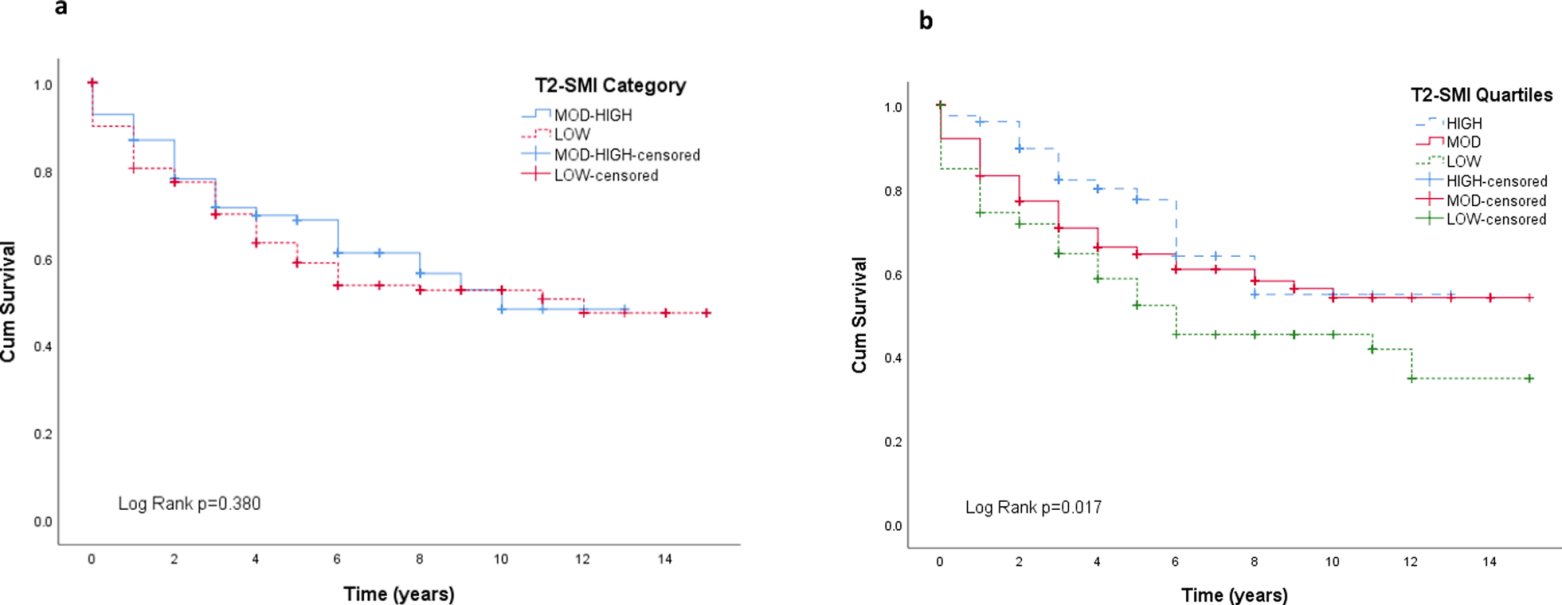
Overall survival **a** T2-SMI categories, **b** T2-SMI quartiles

Kaplan–Meier survival curves with T2-SMI categories did not show a significant difference in survival in those with low T2-SMI (Fig. 3). The lowest 25th percent quartile for the cohort was 63 cm^2^/m^2^, and the 75th quartile was 94cm^2^/m^2^. The OS curve comparing three quartile groups (low, moderate and high) is also shown in Fig. 3, demonstrating that the low threshold of 63 cm^2^/m^2^ in the whole cohort, regardless of sex or BMI, was associated with worse OS (Log Rank *p* = 0.017). This translated to a 1-year survival rate of 73% vs 84% (moderate T2-SMI quartile) vs 95% (high T2-SMI quartile), and 5-year rate of 56% vs 70% vs 81%. Median survival in the low T2-SMI quartile was 7.6 vs 9.6 vs 9.0 years (95% CI 6.17–9.1).

In the sub-set of patients who completed RT, cox regression analysis indicated that on a univariate level, several patient and disease factors were associated with OS, including sarcopenia and low quartile T2-SMI. However, on multivariate analysis, only males, increasing age, and later stage disease (T3, T4 and N3) retained significant association with OS (Table 4).

**Table 4:** Cox regression overall survival analysis

Characteristic	Overall survival				
	*n*	Univariate		Multivariate	
		HR (95% CI)	*p* value	HR (95% CI)	*p* value
Sex					
Female	55	Ref		Ref	
Male	280	1.62 (1.05–2.50)	**0.030**	1.81 (1.15–2.83)	**0.010**
Age (years)		1.04 (1.02–1.06)	**< 0.001**	1.05 (1.03–1.07)	**< 0.001**
Tumour site					
Larynx	53	Ref			
Oropharynx	187	0.87 (0.53–1.41)	0.562		
Nasopharynx	36	0.71 (0.34–1.50)	0.366		
Hypopharynx	15	1.60 (0.71–3.59)	0.258		
Oral cavity	40	1.50 (0.83–2.70)	0.183		
Tumour stage					
T1	96	Ref		Ref	
T2	98	1.49 (0.84–2.65)	0.173	1.43 (0.80–2.55)	0.230
T3	82	2.63 (1.54–4.50)	**< 0.001**	2.65 (1.55–4.56)	**< 0.001**
T4	52	4.01 (2.29–7.02)	**< 0.001**	4.51 (2.56–7.96)	**< 0.001**
Nodal stage					
N0	92	Ref		Ref	
N1	88	0.68 (0.40–1.15)	0.15	0.94 (0.55–1.62)	0.834
N2	136	0.96 (0.63–1.47)	0.863	1.48 (0.95–2.32)	0.085
N3	19	2.90 (1.5–5.59)	**0.002**	4.78 (2.4–9.50)	**< 0.001**
Treatment modality					
RT only	75	ref			
RT + surgery	90	1.40 (0.87–2.26)	0.170		
CRT	170	0.81 (0.51–1.30)	0.387		
BMI category (kg/m^2^)					
Underweight (BMI < 20)	19	Ref		Ref	
Normal weight (BMI ≥ 20 < 25)	99	0.63 (0.34–1.17)	0.145	1.09 (0.56–2.12)	0.801
Overweight (BMI ≥ 25 < 30)	138	0.43 (0.23–0.80)	**0.007**	0.72 (0.37–1.38)	0.320
Obese (BMI > 30)	79	0.46 (0.23–0.90)	**0.024**	0.80 (0.39–1.65)	0.551
Sarcopenia					
No	174	Ref		Ref	
Yes	161	1.47 (1.02–2.10)	**0.037**	0.96 (0.61–1.50)	0.845
Sarcopenic obesity					
No	228	Ref			
Yes	107	0.93 (0.63–1.36)	0.697		
T2-SMI cutoff categories*					
Mod–high T2-SMI	162	Ref			
Low T2-SMI	173	1.14 (0.80–1.64)	0.470		
T2-SMI quartile categories*					
Mod–high quartile	256	Ref		Ref	
Low quartile	79	1.59 (1.09–2.32)	**0.016**	1.08 (0.66–1.75)	0.772

All significant values are in bold (*p* < 0.05)

*RT* radiotherapy, *CRT* concurrent chemoradiotherapy, *BMI* body mass index, *SMI* skeletal muscle index, *HR* hazard ration, *CI* confidence interval

*Tested separately in cox regression models independently with other variables

Patients who presented without sarcopenia had a significantly higher mean percentage weight loss during treatment (6.2% vs 4.9%, *p* = 0.023). This was also observed in those patients with high T2-SMI (6.3% vs 4.9%, *p* = 0.014), and in the higher T2-SMI quartile ranges (3.6% vs 5.7% vs 7.2%, *p* < 0.001). Sixty percent of these patients experienced CWL (*n* = 202), with a third of these presenting with baseline sarcopenic obesity (*n* = 67). A significantly higher proportion of patients with mod–high T2-SMI experienced CWL (*p* = 0.011). Reactive enteral feeding tubes were required in 31% of patients experiencing CWL, in a significantly higher proportion than the rest of the cohort (*p* < 0.001). T2-SMI and weight loss category patient characteristics are shown in Table 5. ROC curves were tested with actual L3 measures, with OS and CSS curves displayed in supplementary material (Figs. 5, 6).

**Table 5:** T2-SMI and weight loss category characteristics in patients who completed RT

	T2-SMI		*p* value	CWL		*p* value
	Low	Mod–high		< 5% loss	≥ 5% loss	
	N = 173	N = 162		N = 133	N = 202	
	(%)	(%)		(%)	(%)	
Sex			0.099			0.961
Male	139 (80)	141 (87)		111 (83)	169 (84)	
Female	34 (20)	21 (13)		22 (17)	33 (16)	
Age (years)			0.684			**0.035**
Mean (SD)	60 (12)	60 (10)		62 (12)	59 (11)	
Tumour site			0.153			**< 0.001**
Larynx	25 (15)	28 (17)		38 (29)	15 (7)	
Oropharynx	94 (54)	95 (59)		52 (39)	137 (68)	
Nasopharynx	20 (12)	16 (10)		7 (5)	29 (15)	
Hypopharynx	12 (7)	3 (2)		11 (8)	4 (2)	
Oral cavity	22 (12)	18 (11)		25 (19)	15 (7)	
Unknown primary	0	2 (1)		0	2 (1)	
Tumour stage			0.210			**0.015**
Tis	1 (1)	2 (1)		1 (1)	2 (1)	
T1	53 (30)	43 (27)		33 (25)	63 (31)	
T2	45 (26)	52 (33)		32 (24)	66 (32)	
T3	45 (26)	37 (23)		34 (25)	48 (24)	
T4	29 (17)	23 (14)		32 (24)	20 (10)	
Tx	0	4 (2)		1 (1)	3 (2)	
Nodal stage			0.234			**< 0.001**
N0	54 (31)	38 (24)		55 (41)	37 (18)	
N1	48 (28)	40 (25)		38 (29)	50 (25)	
N2	62 (36)	74 (45)		35 (26)	101 (50)	
N3	9 (5)	10 (6)		5 (4)	14 (7)	
Treatment modality			**0.017**			**< 0.001**
RT only	48 (28)	27 (17)		43 (32)	32 (16)	
RT + surgery	49 (28)	41 (25)		63 (47)	27 (13)	
CRT	76 (44)	94 (58)		27 (20)	143 (71)	
BMI category (kg/m^2^)			**< 0.001**			**< 0.001**
Underweight (BMI < 20)	15 (9)	5 (3)		15 (11)	5 (3)	
Normal weight (BMI ≥ 20 < 25)	52 (30)	46 (28)		52 (39)	46 (23)	
Overweight (BMI ≥ 25 < 30)	93 (53)	47 (29)		47 (36)	93 (46)	
Obese (BMI > 30)	13 (8)	64 (40)		19 (14)	58 (28)	
Sarcopenia			**< 0.001**			**< 0.001**
Yes	133 (77)	28 (17)		76 (57)	86 (43)	
No	40 (23)	134 (83)		57 (43)	116 (57)	
Sarcopenic obesity*			0.509			**< 0.001**
Yes	87 (65)	20 (71)		40 (53)	67 (78)	
No	47 (35)	8 (29)		36 (47)	19 (22)	
Feeding tubes			0.134			**< 0.001**
No tube	116 (67)	94 (58)		107 (80)	103 (51)	
Prophylactic	28 (16)	28 (17)		18 (14)	37 (18)	
Reactive	29 (17)	41 (25)		8 (6)	62 (31)	
T2-SMI category						
Low	–	–		80 (60)	93 (46)	**0.011**
Mod–high	–	–		53 (40)	109 (54)	

All significant values are in bold (*p* < 0.05)

*SD* standard deviation, *RT* radiotherapy, *CRT* concurrent chemoradiotherapy, *BMI* body mass index, *SMI* skeletal muscle index, *CWL* critical weight loss, *Tis* tumour in situ, *Tx* unknown primary

*In patients with sarcopenia

## Discussion

This is the first study to our knowledge to investigate the use of skeletal muscle at T2 for the development of SMI cutoff values for CT-defined sarcopenia to determine association with OS and weight loss outcomes in patients with HNC. We present sex and BMI-specific cutoff values of T2-SMI for sarcopenia risk stratification in patients with HNC that can be applied when the L3 landmark is not available.

Although the sex and BMI-specific T2-SMI cutoff values did not translate into a significant association with OS, in contrast to the predicted sarcopenia measures, survival was lower in those below the set cutoff values. This is consistent with Pai et al. who also found no relation [21]. There was an association with worse OS in those patients below the 25th percentile threshold regardless of sex or BMI. Quartile thresholds, however, should be used with caution as they may not be a clear indication of true prognostic value [25]. Prediction modelling demonstrated T2-SMI to be predictive of sarcopenia in our cohort when applying our cutoff values, suggesting they are useful in identifying patients at risk of sarcopenia, and can be applied clinically as part of overall patient nutritional assessment. Patients with a T2-SMI below 63 cm^2^/m^2^ may be at the greatest risk with the demonstrated association with OS.

Our findings on the association of sarcopenia and OS are consistent with the two recent meta-analyses in patients with HNC. Wong et al., demonstrated CT-defined sarcopenia was associated with a significantly worse OS in eight studies [14] and Findlay et al., identified that pre and post-treatment sarcopenia were both independent poor prognostic indicators in patients undergoing radiotherapy treatment in six studies [13]. Of note, however, is the heterogeneity of tumour sites used in these studies and the varying vertebral levels and sarcopenia cutoff values used.

The T2-SMI cutoff values in the present study were determined with ROC curves based on sarcopenia assessment using predicted L3-SMI, with BMI and sex stratifications. The sarcopenia cutoff values suggested by Martin et al. [24] applied for this categorisation are not based on a HNC population. As specific cutoff values for sarcopenia in HNC have yet to be established, these were deemed the most closely representative of our population and have been previously applied in an Australian cohort [26]. However, this may have an impact on our final findings. We note that those cutoff values are survival-based, and generated from a heterogeneous sample of patients with respiratory and gastrointestinal tumours, who have different survival rates, and may impact on the outcomes of our study. Results may also have been influenced by the percentage error in the prediction of values at L3. However, testing conducted on the sample cohort using actual L3 measures found similar corresponding T2-SMI cutoff values and the AUC was high in all groups indicating good discriminative power for the low T2-SMI values.

Outcome-based low skeletal muscle cutoff values for patients with HNC, e.g., risk of toxicity, dysphagia, post-operative complications, survival etc., have been suggested by several authors, mostly using the C3 vertebral landmark and presenting cutoff values in terms of predicted measures at L3. However, several limitations should be noted in these studies, namely, that; most lack sex-specific stratification [27–31], or have used population-specific median, mean or percentile values [21, 32–34], or have been extracted from populations that differ in ethnicity [21, 28, 35] (and, therefore, average body sizes/BMIs), deeming suggested cutoff values difficult to transfer across populations. Recently, Zwart et al. suggested sex-specific low C3-SMI values for risk of radiotherapy toxicities in a similar, yet smaller cohort (*n* = 196), applying the ROC curve method; however, all AUC values were poor at < 0.7 [36]. Similarly, Galli et al. applied ROC curves for low cervical SMM cutoff values, again with low AUCs < 0.63 [37]. The difficulty with establishing universal cutoff values in HNC is the heterogeneity of patients, with differing presentation characteristics for each specific tumour site, and studies investigating different cohorts of tumours of the head and neck (e.g., inclusion of salivary gland or nasal cavity tumours). Survival also differs, especially with the inclusion of patients with small, early stage laryngeal tumours, and oropharyngeal carcinomas, as those with human papillomavirus-positive disease, for example, demonstrate better survival rates [38]. Toxicity-related weight and muscle loss can also be tumour-site dependent as treatment varies; therefore, sarcopenia cutoff values for HNC may be more applicable if they were sex, ethnicity, and tumour site-specific.

In several studies using C3, cutoff values are provided as a predicted value at L3, via the application of the Swartz et al. [39] prediction model [27, 29, 36]. Our group has demonstrated this method lacked clinically acceptable agreement with L3 measures in our population [18], and therefore, we applied our prediction model for T2-CSA conversion in this study [20]. The ROC curves generated in the present study using our predicted measures and T2-SMI had high sensitivity and specificity levels of over 70%. This demonstrates that SMI measures derived from skeletal muscle at T2 are a feasible option for muscle mass assessment in this population.

Thoracic skeletal muscle assessment has been investigated previously in HNC. Pai et al. (*n* = 398) used median T2-SMI sex-specific cutoff values which were considerably lower than found in our study; females < 34.3 cm^2^/m^2^ vs < 74 cm^2^/m^2^ and males < 51.74 cm^2^/m^2^ vs < 66 cm^2^/m^2^ and < 88 cm^2^/m^2^) [21]. Matsuyama et al. suggested a prediction model using T12 in a Japanese population [40], deemed unsuitable to apply to our cohort due to the differences in ethnicity and T12 not being visible in a head and neck scan. We chose T2 as this musculature is associated with muscle wasting, and is assessed as part of the physical examination of the Subjective Global Assessment, used to diagnose malnutrition [41, 42]. This level is visible in a head and neck CT scan and a radiotherapy planning scan, increasing the accessibility for clinical application.

CWL loss during treatment has been shown to negatively impact both patient survival and the ability to tolerate treatments [3, 43, 44]. We have demonstrated that patients with higher T2-SMI experienced significantly higher percent weight loss during treatment. Our Australian cohort is typical of a western population, having the majority of patients classified as overweight or obese. Approximately two-thirds of the cohort in this study presented as overweight or obese, and 70% of these patients lost ≥ 5% of their body weight during radiotherapy (the majority having a mod–high T2-SMI). This could explain the difference in weight loss, as patients who present as overweight or obese, may not have early nutritional intervention if there are no visible signs of wasting or malnutrition, due to a misconception that they have adequate muscle stores. Reactive feeding tubes are often inserted when high percentage weight loss has occurred, and in this cohort, more patients with mod–high thoracic muscle stores required this intervention.

Prado et al. demonstrated sarcopenia in obese patients is independently associated with worse survival [45], and without body composition assessment prior to treatment commencement for HNC, high risk patients may be overlooked for early interventions. A small proportion of patients who were either overweight or obese and sarcopenic (12%), had high T2-SMI measures. This represents a cohort of patients for which the application of the T2-SMI cutoff values in this study alone may not identify all patients with sarcopenic obesity. Nutritional assessments in clinical practice should incorporate several screening measures, as one parameter alone may not be an indicator of nutritional risk.

This small, single-centre retrospective study does have some limitations. The number of patients excluded due to “unusable” scans was considerable, and may have impacted on overall findings and created a bias. Ideally, prospective scan analysis, ensuring that all muscles at T2 are visible and patient positioning is optimum, would enable more robust investigation. The T2-SMI cutoff values have been determined through the application of a prediction model to estimate L3-SMI for sarcopenia stratification, and should be validated against true measures at L3. However, at our Cancer Centre, as with many internationally, it is not standard practice for all patients with HNC to have a PET–CT scan that would have L3 visible. Consequently, this adds to a cohort number that, although substantial, may not have been adequate to demonstrate definitive outcomes with T2-SMI application. HNC patient numbers are relatively small in Australian Centres, and a multi-centre collaborative approach is required for future research and validation of T2. The heterogeneous HNC sites in this cohort should also be considered and may affect applicability across populations. Tumour-site specific SMI thresholds are likely required. This study used CT muscle measures to define sarcopenia. Measures of muscle strength and function should ideally be incorporated into future research for thorough investigation of muscle loss outcomes in HNC.

In this study, we have suggested sex and BMI-specific T2-SMI cutoffs that are associated with sarcopenia in patients with HNC, and also a low T2-SMI value from the lowest quartile of the cohort to identify those at highest risk of worse OS.

## Conclusions

T2 is a clinically relevant, readily accessible vertebral level in a head and neck CT scan or radiotherapy planning scan, and provides a larger group of skeletal muscles to assess for depletion in patients with HNC. Application of the suggested cutoff values for sarcopenia detection can be used for risk screening and appropriate and timely nutritional intervention.

### Supplementary Information

Below is the link to the electronic supplementary material.

Supplementary file1 (DOCX 6389 KB)

## Data Availability

No additional data are available.
