# Supplementary material for: Thoracic skeletal muscle index is effective for CT-defined sarcopenia evaluation in patients with head and neck cancer
Source: Eur Arch Otorhinolaryngol. 2023 Aug 12;280(12):5583–94. doi: 10.1007/s00405-023-08162-y (PMC10620319; doi:10.1007/s00405-023-08162-y)
Supplement: Supplementary file 1 — Supplementary file1 (DOCX 6389 KB) [file 405_2023_8162_MOESM1_ESM.docx]

**SUPPLEMENT FIGURES (testing with actual L3)**


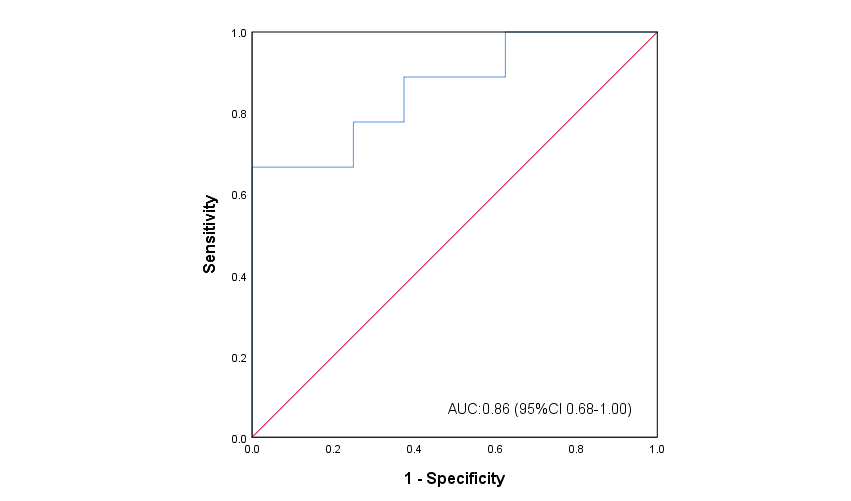

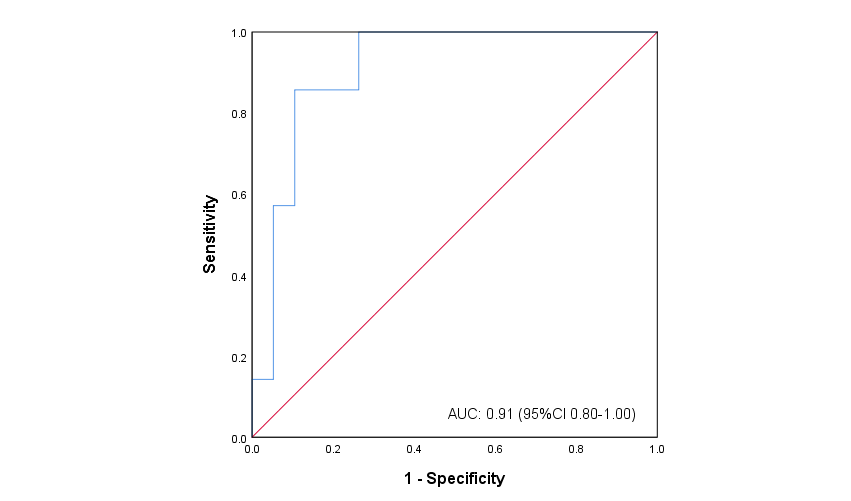

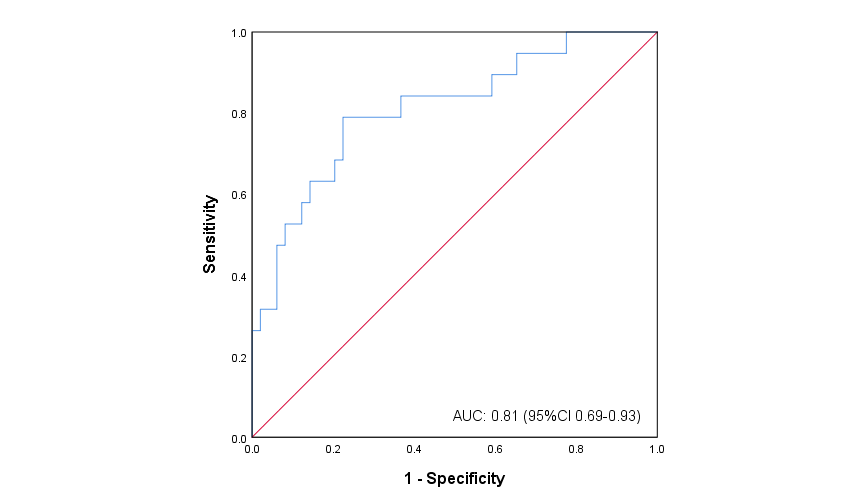


**a**

**b**

**Fig 4.** a) Females b) Males BMI<25kg/m^2^ and BMI≥25kg/m^2^

**
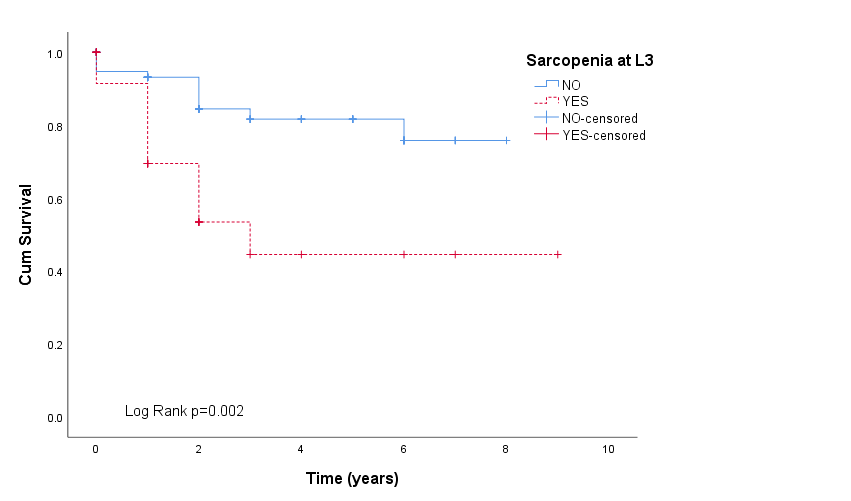
Fig 5**. OS with actual L3 measures

**
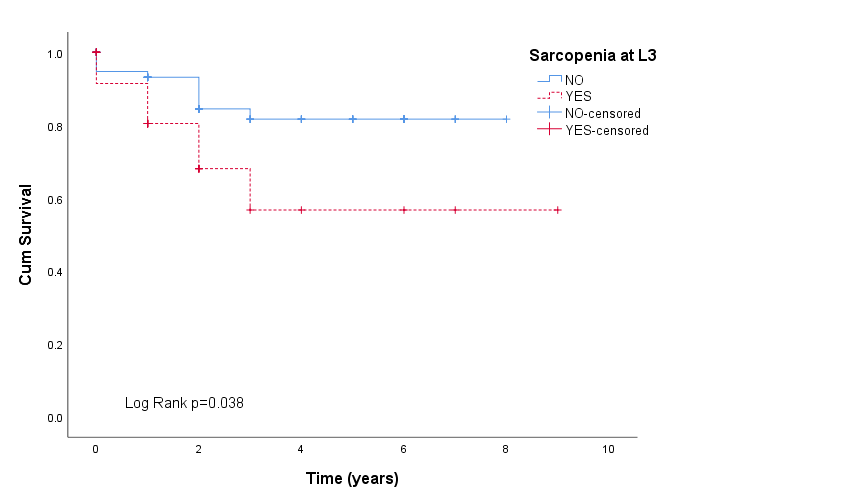
Fig 6.** CSS with actual L3 measures
